# Thermal Manipulation During the Embryonic Stage and the Post-Hatch Characteristics of Broiler Chickens

**DOI:** 10.3390/ani14233436

**Published:** 2024-11-27

**Authors:** Ana Patrícia Alves Leão, Alexandre Vinhas de Souza, Daniella Rabelo Barbosa, Carina Fernanda Gomes da Silva, Renata Ribeiro Alvarenga, Itallo Conrado Sousa de Araújo, Adriano Geraldo, Carla Oliveira Resende, Márcio Gilberto Zangeronimo

**Affiliations:** 1 Department of Animal Science, Federal University of Lavras, Lavras 37202-203, MG, Brazil; anapatriciaalvesleao@gmail.com (A.P.A.L.); alexandre.vinhas.souza@gmail.com (A.V.d.S.); daniellarabelo@outlook.com (D.R.B.); carinagms29@gmail.com (C.F.G.d.S.); renata.alvarenga@ufla.br (R.R.A.); carla.reo@hotmail.com (C.O.R.); 2 Department of Animal Science, Federal University of Minas Gerais, Belo Horizonte 31270-901, MG, Brazil; italloconradovet@hotmail.com; 3 Department of Animal Science, Federal Institute of Minas Gerais, Bambuí 38900-000, MG, Brazil; adriano.geraldo@ifmg.edu.br; 4 Department of Veterinary Medicine, Federal University of Lavras, Lavras 37202-203, MG, Brazil

**Keywords:** egg incubation, fattening chicken, heat stress, poultry farming, temperature, thermotolerance

## Abstract

The objective of this study was to evaluate the influence of thermal manipulation during incubation on the hatchability, the performance, the carcass characteristics, the intestinal villi, the nutrient metabolizability, and some physiological parameters in broiler chickens. Ross eggs were randomly distributed into four commercial automatic incubators. The treatments were as follows: incubator kept at 37.5 °C throughout the incubation period (Ctrl—control), and incubator temperature increased to 39 °C on days 16, 17, and 18 of incubation for 3 (T_3h_), 12 (T_12h_), or 24 h (T_24h_). The chicks were housed in an experimental performance barn and kept until 42 days of age under standard rearing conditions. The hatchability was lower when T_24h_ was used. During the time after hatching, T_3h_ caused higher levels of heat shock protein 70 (HSP70) in the liver, a faster breathing rate, and a drop in the cloacal temperature and hematocrit. T_12h_ increased the respiratory rate. T_24h_ decreased the hematocrit, the weight gain, and the feed intake. The thermal manipulation during incubation did not affect the carcass characteristics, intestinal villi, or metabolizability of nutrients. It was concluded that raising the incubator temperature for 3 h during late incubation may be beneficial for thermoregulation, but raising it for 24 h during this period impairs animal performance.

## Simple Summary

Heat stress negatively affects broiler chicken production, particularly in high-performance breeds. Researchers have shown that performing thermal manipulation during incubation can improve the birds’ thermotolerance and minimize the negative effects of heat. This study evaluated the influence of increasing the temperature to 39 °C for 3, 12, or 24 h in the last phase of incubation on the hatchability, post-hatch performance, metabolism, and behavioral and physiological changes in broiler chickens. The results of this study revealed that increasing the incubation temperature to 39 °C for 3 h did not affect the performance or carcass characteristics, but improved the birds’ responses to heat during the rearing phase. However, applying 39 °C for 24 h reduced the birds’ performance in the rearing phase. In conclusion, increasing the temperature to 39 °C for 3 h during the late incubation stage may be beneficial for the thermoregulation of broiler chickens.

## 1. Introduction

High ambient temperatures are predicted to occur more frequently in the years to come due to global warming, which presents serious difficulties for poultry production, particularly in tropical areas [1]. One of the main negative effects of heat stress on meat chickens is that contemporary broiler genetic lines, which are distinguished by their quick growth and significant muscle mass gain, have higher metabolic activity as a result. The ability of these birds to effectively dissipate heat is limited, though, by the slower development of visceral organs, such as those of the cardiorespiratory system [1,2]. Birds naturally limit their feed intake to reduce metabolic heat generation when ambient temperatures rise above the thermal comfort zone [3], and this increases the amount of metabolic energy needed to maintain homeostasis [4]. As a result, feed conversion deteriorates, weight gain declines, and illness susceptibility rises, ultimately resulting in financial losses for producers [5]. Completely reducing heat stress is still a significant issue, even in climate-controlled buildings [6].

The incubation time represents 1/3 of the lifespan of broilers. Thus, understanding the relationship between the embryonic development period and the post-hatch performance of broilers is important [7]. Among the factors influencing embryogenesis, the incubation temperature is the main limiting factor. In commercial hatcheries, the incubator air temperature considered optimal for embryonic development varies between 37.5 and 37.8 °C, and the ideal eggshell temperature (EST) ranges between 37.8 and 38.3 °C [8,9]. Thermal manipulation (TM) during incubation is a strategy to mitigate the detrimental effects of heat stress on birds [10]. This technique consists of raising the temperature for short periods during a certain phase of embryonic development. The objective is the acquisition of thermotolerance in birds after hatching. One study suggests that TM is related to epigenetic changes [11]. However, the development of thermotolerance via TM depends on several factors, such as the embryonic age, temperature variation, and duration of manipulation.

Amjadian and Shahir [12] suggest that it should align with the maturation of the embryonic thermoregulatory system. According to Piestun et al. [13], this development occurs between the 6th and the 16th days of incubation. Morita et al. [14] evaluated three constant incubation temperatures (36.0, 37.5, and 39.0 °C) from the 13th day until hatching. The eggshell temperatures were 37.4 ± 0.08 °C, 37.8 ± 0.15 °C, and 38.8 ± 0.33 °C, respectively. The authors observed that the broilers from eggs incubated at 39.0 °C preferred warm environments and had lower plasma T3 concentrations during the post-hatching period. The researchers did not evaluate the hatchability, and they observed no impact on broiler performance. Al-Zghoul et al. [15] reported an increased performance and muscle mRNA levels of heat shock protein 70 (HSP70) in the chicks from eggs subjected to 38.8 °C for 18 h during the 10th–18th days of incubation. The authors suggested that manipulating egg incubation conditions may improve the tolerance to heat stress in chickens. However, the effects on the hatchability and survival of birds after hatching have made it difficult to use thermal manipulation programs in commercial hatcheries. In fact, most studies have reported a reduction in hatchability [16,17,18,19,20]. Thus, the objective of the present study was to evaluate the influence of TM applied on days 16, 17, and 18 of incubation on the hatching, performance, and thermotolerance in broilers.

## 2. Materials and Methods

### 2.1. Location

This study was carried out in the Poultry Sector of the Department of Animal Science at the Federal University of Lavras (UFLA), located in Lavras, MG, Brazil. The Ethics Committee on Animal Use of the University approved all the procedures under protocol number 028/18.

### 2.2. Incubation

All the procedures described below were repeated successively 4 times. In each repetition, 280 Ross 308 eggs (1120 in total) from 30–42-week-old breeders were obtained from a commercial hatchery. Upon arrival, the eggs were pre-heated to 28 °C for 9 h. Then, the eggs were fumigated with formaldehyde and potassium permanganate at a 2:1 ratio and incubated in an automatic incubator (Luna 480, Chocmaster, Piraquara-PR, Brazil) set to 37.5 °C and 65% humidity. The incubator was placed inside a climate-controlled room at 20 °C, with egg turning scheduled every 2 h.

Candling was performed on the 15th day. After the examination, 216 for each time period (864 in total for the experiment, weighing 57.26 ± 3 g (mean ± SD) with confirmed embryonic development) were randomly and equally distributed into four incubators (Luna 480, Chocmaster, Piraquara-PR, Brazil) with different temperature settings: eggs incubated at a constant temperature (37.5 °C) until the end of incubation (Ctrl—control), and eggs subjected to a temperature of 39.0 °C on days 16, 17, and 18 of incubation for 3 h/day (T_3h_), 12 h/day (T_12h_), or 24 h/day (T_24h_). On Day 19, the temperature of the incubators was decreased to 37.0 °C, and egg turning was interrupted. The relative humidity was maintained at 60% until the end of the incubation period. The thermal treatments were defined based on information obtained in the literature [14,21]. The experimental design was a randomized block design consisting of four treatments and four replicates (time) with 54 eggs each.

On the 16th, 17th, and 18th days of incubation, the shell temperature was measured for 10 randomly selected eggs/treatment/replicate. The measurements were taken through the glass door of the incubator using a thermographic camera (Fluke TiS55, Everett, Washington, DC, USA; resolution of 0.1 and accuracy of 1.5%) at three time points: before, during (half point), and at the end of TM.

The incubation time was determined by recording the hatching of the first bird. The hatching window was calculated based on the time from the hatching of the first bird to the hatching of the last.

After hatching, the live chicks were quantified, weighed, sexed, and vaccinated against Marek’s disease. Eight chicks from each treatment, four males and four females (*n* = 8), were randomly selected and slaughtered for organ weighing (precision analytical balance, ±0.0001 g) and blood collection. The hatchability (hatched birds/total number of eggs at the 15th day of incubation), hatching weight (g), and relative organ weight at hatching (g/g of live weight) were also recorded.

### 2.3. Performance and Carcass Characteristics

After hatching, the chicks were housed in a screened masonry experimental shed. The pens (2.0 × 1.5 m) featured cement floors and were lined with wood shavings, and these pens were considered the experimental units. For each repetition, two pens, each containing 12 birds (6 males and 6 females randomly selected; 4 birds/m^2^) were used for each treatment, resulting in a total of 32 experimental units (4 time replicates). By changing the curtains and heating the lights, the environmental conditions were managed. At 7:00 am and 5:00 pm, a digital thermohygrometer (Simpla, TH02, Asko^®^, São Leopoldo, Brazil) was used to measure the temperature and relative humidity. Table 1 displays the environmental conditions in which the broilers from eggs exposed to TM during incubation were raised. The Ross Broiler Management Handbook’s requirements were adhered to by the management procedures [22].

The experimental feeds were formulated according to the nutritional requirements of high-performance mixed broilers [23] at each rearing stage (Table 2). At the beginning and end of each rearing period, the birds, the feed provided, and the leftovers were weighed to determine the weight gain (g) and feed intake (g). The feed conversion (g/g) was calculated by the feed intake/weight gain ratio.

At 21 and 42 days of age, one bird from each pen (a total of 8 birds/treatment) was selected according to the weight closest to the average weight of the plot, fasted for 6 h, weighed, and slaughtered. The birds were then bled at the jugular artery for blood collection. The carcasses were plucked and eviscerated, the head and feet were removed, and the carcass was weighed. The carcass yield (carcass weight/live weight before slaughter), breast, and thigh + drumstick with bones yield (relative to carcass weight) were evaluated. The lymphoid organs (thymus, spleen, and bursa of Fabricius) were also weighed to determine their weight relative to the live weight.

### 2.4. Blood Parameters

At 1, 21, and 42 days of age, a drop of fresh blood obtained from the slaughtered birds (*n* = 8) was used to measure the glucose concentration using an Accu-Check^®^ kit (Roche, São Paulo, Brazil).

To determine the hematocrit, capillary tubes were filled 2/3 with blood samples and then centrifuged at 3000× *g* for 10 min. Then, using a 30 cm ruler, the percentage of blood occupied by erythrocytes was calculated. The capillary tubes were then sectioned at the boundary between the plasma and the blood elements, and a drop of plasma was deposited on an analog refractometer (RHC-200ATC, Danoplus, Mainland, China) for the determination of the total plasma protein (TPP) in g/dL by refractometry.

For the biochemical tests, a blood sample was also drawn and placed into two thirds of a vacuum tube devoid of anticoagulant. After centrifuging the samples for 10 min at 3000× *g*, the serum was extracted and kept at −80 °C for further examination. ELISA kits (cat. No. E0098Ch and E0022Ch, respectively; Bioassay Technology Laboratory, Xangai, China) were used to test the serum levels of corticosterone and T3.

A blood smear was conducted at 42 days of age in order to determine the heterophil/lymphocyte ratio and differential leukocyte count.

### 2.5. Gene Expression of Heat Shock Protein (HSP70)

At 42 days of age, a liver sample was aseptically collected, placed in a nuclease-free tube, and stored at −80 °C for the determination of the gene expression of HSP70 [21]. The target and reference primers were designed using sequences published in the public GenBank database of the National Center for Biotechnology Information (NCBI) platform (accession numbers: ACTB (NM_205518.1), GAPDH (NM_204305.1), and HSP70 (J02579.1)). The primers were formulated using the OligoPerfect Design software (Invitrogen, Karlsruhe, Germany, https://www.thermofisher.com/ro/en/home/life-science/oligonucleotides-primers-probes-genes/custom-dna-oligos/oligo-design-tools.html, accessed on 24 November 2024) and synthesized (Invitrogen, Carlsbad, CA, USA)—ACTB (F: GATCTGGCACCACACTTTCT R: TCTTCTCTCTGTTGGCTTTGG), GAPDH (F: AGATGCAGGTGCTGAGTATG R: CTGAGGGAGCTGAGATGATAAC), and HSP70 (F: GGATGAAGCCAACAGAGATAGG R: TTGTCCTGGTCACTGATCTTTC). The total RNA was extracted from 50 mg of a liver sample using QIAzol (Qiagen, Valencia, Spain). The isolated RNA was treated with DNA-free DNAse (Ambion, Austin, TX, USA) according to the manufacturer’s instructions. The amount (ng/μL) and quality (260/280 and 260/230) of RNA were evaluated using a spectrophotometer (DeNovix DS-11 Spectrophotometer, USA) at 260 nm. The total RNA was subjected to electrophoresis in a 1.0% (*w*/*v*) agarose gel stained with the GelRed nucleic acid gel stain (Biotium, Hayward, CA, USA). The 28S and 18S rRNA bands were analyzed using an E-gel Imager Camera Hood (Life Technologies, Snow Yamin, Israel) to check for possible degradation. cDNA synthesis was performed according to Ferreira et al. [24].

The RT-qPCR analyses for each examined gene were conducted using cDNA from eight biological replicates (birds), each with two technical replicates. The results were normalized by the ^2−ΔΔ^CT method for the expression of the reference genes β-actin and GAPDH (glyceraldehyde 3-phosphate dehydrogenase). The relative expression levels were calculated according to the method described above based on the ^2−ΔΔ^CT values, which were corrected by the amplification efficiency for each primer pair.

### 2.6. Morphometry of the Intestinal Mucosa

To evaluate the morphometry of the intestinal mucosa, 1 to 2 cm mid-jejunum samples were collected at 21 days of age (*n* = 8). The samples were washed with a saline solution to remove the intestinal contents and were placed in 10% buffered formalin for 24 h. Subsequently, routine histological procedures were applied. First, the samples were dehydrated in an increasing ethanol series, purified in xylene, embedded in paraffin, sliced into 4.0 µm sections, placed on glass slides, and dried at 37 °C overnight. Hematoxylin and eosin were then used to stain the samples.

The images were analyzed using an Olympus microscope (CX31; Olympus, Tokyo, Japan) coupled to an Altra digital camera (SC30, Olympus, Tokyo, Japan) with the ImageJ software v.1.53 [25]. A total of 10 readings/slides were performed. The measurements included the assessment of villus height and crypt depth. The villus height was determined from the villus tip to the junction with the crypt, while the crypt depth was defined as the depth of the invagination between adjacent villi. Subsequently, the villus/crypt ratio was computed.

### 2.7. Thermal Challenge

At 10, 17, 24, and 31 days after hatching, thermal challenge tests were performed [26]. For these tests, four acrylic climate chambers (80 × 80 × 80 cm) equipped with heaters, fans, and humidifiers were used. On each test day, one bird from each experimental pen (*n* = 8) was randomly selected and transported to the Ambience Laboratory of the Engineering Department of the Federal University of Lavras. During the tests, the animals were kept for 45 min at room temperature for adaptation. Subsequently, the chamber temperature was increased by 5 °C for 45 min. After the chamber temperature returned to the initial temperature, the birds remained in this location for another 45 min. The relative humidity was fixed at 60%, and the air velocity was 0.2 m/s throughout the analysis period [27].

Every 45 min of the test (before the challenge, at the end of the challenge, and 45 min after), the cloacal temperature (CT) was measured using a digital thermometer (Joytech Healthcare, Yuhang District, Hangzhou, China, precision of ±0.1 °C), and the respiratory rate (RR) was registered by observing the number of breaths per minute. The observers were blinded to the bird’s experimental group. After the tests, the birds were marked with a marker brush so that they would not be used in the tests in the following weeks.

### 2.8. Metabolizability of Nutrients

For the metabolism test, 128 15-day-old males and females were used. The birds were housed in groups of four (two males and females) in metabolic cages (50 × 50 × 50 cm) with a screened floor and provided with a pressure drinker (1 per cage) and a trough-type feeder located along the entire front extension. The cages were kept in a room equipped with exhaust fans. Each treatment was represented by 8 experimental units (*n* = 8).

From the 15th to the 25th days of age, four birds were kept per cage. From the 25th day onward, one randomly chosen female was removed per cage. The temperature and relative humidity were monitored at 7:00 am and 5:00 pm using a digital thermohygrometer (Simpla TH02, Asko^®^, São Leopoldo, Brazil) located near the cages.

The excreta were collected at 22, 23, and 24 days and at 35, 36, and 37 days of age, always in the morning, following a previously described method of total excreta collection [28]. The feed was kept in buckets labeled with the cage number. Twenty-four hours before the beginning of the collection period, the amount of feed in the buckets was weighed, and the feeders were emptied and immediately replenished with the weighted feed. At the end of the collection period, the feeders were emptied again, and the leftovers (feeder + bucket) were weighed to determine the feed intake.

All foreign material, such as feathers and feed particles, were removed from the excreta before the samples were placed in previously labeled plastic bags, weighed, and stored in a freezer until the end of the experimental period, when they were thawed and homogenized. The samples of excreta were processed according to Neves et al. [29].

The samples of feces and feed were analyzed for their dry matter (DM), ash, ether extract, and nitrogen (N) contents, following the techniques described by Silva and Queiroz [30]. Based on the laboratory results, the metabolizability coefficients of the dry matter, protein, and ether extract of the feeds were calculated according to the equation described by Matias et al. [31]:

$$\mathrm{Metabolizability}\;\mathrm{of}\;\mathrm{nutrients}\;(\%)=\frac{\mathrm{Nutrient}\;\mathrm{ingested}\left(g\right)-\mathrm{Nutrient}\;\mathrm{excreted}\left(g\right)}{\mathrm{Nutrient}\;\mathrm{ingested}\left(g\right)}\times 100$$

### 2.9. Statistical Analysis

The hatchability was analyzed using the generalized linear model (GLM) for binomial distribution with the logit function [32]. The other variables were subjected to normality and homoscedasticity of variance tests and then subjected to an analysis of variance (ANOVA). When the ANOVA assumptions were not met, the data were log-transformed. In the case of significant differences, the means were compared by the Scott-Knott test at a 5% probability. A randomized block design with four treatments and eight replicates was used. For the respiratory rate and body temperature during the thermal challenge test, a randomized block design with a 4 × 3 factorial arrangement (four treatments and three evaluation times—before, during, and after the thermal challenge) was used. All the statistical analyses were performed using the STATA 16.0 software.

## 3. Results

### 3.1. Hatching Characteristics

A lower hatchability (*p* < 0.05) was observed in the T_12h_ and T_24h_ compared to the Ctrl and T_3h_ treatments. There was no effect (*p* > 0.05) of TM on the incubation time, hatching window, hatching weight, blood parameters, or relative organ weight in newly hatched birds (Table 3).

### 3.2. Performance

At all the evaluated ages (Table 4), T_24h_ reduced (*p* < 0.05) the feed intake. A lower weight gain (*p* < 0.05) was observed at 1 to 42 days of age. There was no effect of TM during incubation on the feed conversion (*p* > 0.46) or body weight (*p* = 0.14).

### 3.3. Blood Parameters, Organ Weight, Carcass Characteristics, and Gene Expression in the Liver

There was no effect (*p* > 0.19) of TM during incubation on the blood parameters, relative weight of organs, or morphometry of the intestinal mucosa of birds at 21 days of age (Table 5). At 42 days of age, T_3h_ and T_24h_ reduced (*p* < 0.05) the hematocrit. A higher (*p* < 0.05) gene expression of HSP70 in the liver was observed in the birds from eggs incubated under the T_3h_ program. There was no effect (*p* > 0.12) of TM on the carcass characteristics of broilers.

### 3.4. Thermal Challenge

Thermal manipulation during egg incubation did not influence (*p* > 0.42) the respiratory rate of the birds subjected to the thermal challenge at 10, 17, or 24 days of age (Table 6). At 31 days, a higher (*p* < 0.05) respiratory rate was observed in the birds from eggs subjected to T_3h_ and T_12h_ before, during, and after the thermal challenge.

Regarding the cloacal temperature, lower values (*p* < 0.05) were observed for T_3h_ before, during, and after the thermal challenge of the 24-day-old birds (Table 7). There was no effect (*p* > 0.09) of the TM during incubation on the cloacal temperature at the other evaluated ages.

### 3.5. Metabolizability of Nutrients

There was no effect (*p* > 0.33) of TM during incubation on the metabolizability of dry matter, ash, lipids, or proteins in the rations when evaluated at 24 and 37 days of age (Table 8).

## 4. Discussion

To date, there is no agreement on the optimal TM for improving the post-hatch performance of broilers, which poses a challenge for the implementation of an effective program in commercial hatcheries. Furthermore, many studies have reported losses in hatchability with an increasing incubation temperature at different times of embryonic development [20,33,34]. The results of the present study indicate that a temperature of 39 °C for 24 h/day on Days 16, 17, and 18 impaired the hatchability and post-hatch performance of broilers. On the other hand, increasing the incubation temperature by smaller periods (3 or 12 h/day) did not influence the hatchability or the broiler performance, but seemed to have some positive influence on the ability of the birds to respond to thermal variations during the day.

The thermal challenge that broilers face during rearing is essential in highlighting the impact of thermotolerance on their performance. According to Curtis [35], the thermal comfort zone of adult birds ranges from 15 to 28 °C. The maximum temperatures observed in the present study (Table 1) reveal that the birds faced a thermal challenge during the day. However, the brief physiological changes induced by TM during incubation may not have significantly impacted the broiler’s performance. Khaleel et al. [36] observed that 39 °C for 18 h/day from the 7th to the 16th days of incubation enhanced the body weight of the broilers at 35 days of age, reared under normal conditions during the growth phase. The birds maintained this same result when subjected to heat stress (35 °C) from 21 to 28 days of age. The authors reported that these results were due to the intestinal epithelium being more intact in birds that hatched from eggs that had been heated or cooled during incubation. Environmental conditions significantly influence the morphology of the small intestine [37]. A normal morphology as well as the integrity of the intestinal epithelium are of paramount importance for the efficiency of digestion and nutrient absorption processes [38]. Cellular responses to heat stress involve the HSPs in maintaining the integrity of structural proteins [39]. Previous studies have shown that thermal stress may affect the integrity of the intestine, which then causes pro-inflammatory cytokines to rise [38,40,41]. However, these studies took place during the rearing phase. There is evidence that the production of HSPs during the period of embryonic development may also influence the intestinal integrity after hatching [36]. In the current study, the short TM during the final third of incubation did not affect the intestinal integrity of the broilers, leading to non-significant differences in their ability to utilize the nutrients in their diets. This result may also account for the absence of significant improvements in the animals’ performance.

Regarding performance, most studies that have evaluated TM during incubation have observed negative or non-significant results [19,34,42,43]. The amplitude of the temperature and the duration of the TM may both influence these results. Abuoghaba [42] observed a decrease in broiler weight gain when he increased the temperature from 37.5 to 40 °C for 3 h a day between the 6th and 8th days of incubation. The author attributed these results to the variability in the chick’s initial body weight, which reflected the final body weight. Meteyake et al. [19] also noted a decrease in performance when they increased the incubation temperature from 37.5 to 39.5 °C for 12 h a day between Days 7 and 16 of incubation. The authors attributed this result to greater egg weight loss during incubation. According to Swann and Brake [44], the relative humidity has a highly significant effect on egg weight loss during incubation. Meteyake et al. [19] believe that a relative humidity of 65% is excessive for Ross eggs. According to Piestun et al. [45], these conditions may pose a significant challenge for embryos in dissipating excess heat, potentially leading to growth retardation. In that case, an adjustment to the relative humidity may be necessary in extended TM programs, such as the T_24h_ used in the present study.

On the other hand, Tarkhan et al. [33] observed positive results for the weight gain of broilers when they raised the temperature to 39 °C for 18 h a day between the 10th and 18th days of incubation. The authors explained that broilers exposed to TM during incubation had faster myoblast growth and heavier pectoral muscles [46]. According to Piestun et al. [47], growth factors such as insulin-like growth factor I (IGF-I) induced by TM during embryonic development can be associated with muscle cell proliferation and muscle hypertrophy. These authors raised the incubation temperature of Cobb eggs to 39.5 °C (65% relative humidity) for 3 or 6 h daily, starting from Embryonic Days 16 to 18.

In general, animals can respond to heat challenges. When birds are in environments with temperatures above their thermal comfort range, behavioral and physiological changes occur to facilitate heat dissipation or minimize their metabolic production [5]. At high temperatures, the efficiency of heat loss relative to sensitive processes decreases. Therefore, it becomes necessary to utilize alternative pathways such as an increased respiratory rate, which promotes heat loss through evaporation, or a reduced concentration of thyroid hormones [48]. Thyroid hormones are most important for thermogenesis control, and a reduction in T3 levels may be a mechanism for reducing metabolic heat production [14,49]. In non-adapted birds, there is an increase in corticosterone levels [50], which play an important role in metabolic regulation, particularly in reducing feed intake. While thermal stress may have occurred during the rearing phase in the present study, it may not have been sufficient to increase the thyroid hormones and corticosterone levels.

Regarding the thermal challenge, studies have shown that TM during the embryonic phase can favor the rapid adaptation of post-hatching birds to this challenge [14,50]. In this research, we observed that broilers from eggs that had been treated with T_3h_ or T_12h_ had a higher respiratory rate when they were put through the thermal challenge test compared to the Ctrl and T_24h_ treatments. This rate stayed higher until the end of the test. This result suggests a rapid adaptation of these birds to adverse conditions.

Hematological parameters can be used to assess physiological responses to heat stress [51]. Blood plays a key role in thermoregulation, transferring heat from the visceral organs to the body’s periphery [52]. Hemodilution, a process associated with increased blood flow due to peripheral vasodilation, may be responsible for the lower hematocrit values observed in the T_3h_ and T_24h_ treatments at 42 days [51,53]. This response could indicate an adaptation mechanism to enhance heat dissipation. Petrolli et al. [54] suggest that lower hematocrit levels may serve as an indicator of physiological adaptation to heat stress in birds. The T_12h_ treatment, on the other hand, did not significantly change the hematocrit compared to the control. This suggests that the moderate thermal change may not have caused the same level of hemodilution or adaptive stress response. Similarly, Altan et al. [55] reported no significant differences in the hematocrit of broilers subjected to acute heat stress at 44 days of age.

Environmental factors during embryonic development may influence the specific epigenetic profile of tissues and the responses to stressors. In fact, several studies have evaluated the effects of TM, both in the initial phase [13,56] and in the final half of embryonic development [12,57]. Baarendse et al. [58] and Al-Zghoul et al. [15] suggest that raising the temperature during the incubation process might help the cells become used to higher temperatures by changing the levels of thyroid hormones and corticosterone and the expression of HSP70. In the present study, we observed that broilers from eggs subjected to T_3h_ had a higher expression of HSP70 in the liver. This result suggests that these birds may be more capable of adapting to adverse temperature conditions. In the case of broilers subjected to the thermal challenge, handling them at the site of the challenge may have raised their body temperature, resulting in a better physiological response than the other birds.

However, studies investigating TM during incubation have often observed a reduced hatchability and bird survival after hatching [16,17,18,19,20]. It is known that the internal egg temperature directly influences embryonic development, which in turn affects the hatchability [59]. In the present study, the use of T_12h_ or T_24h_ reduced the hatchability. Maintaining a temperature of 39 °C for 24 h (T_24h_) impaired the birds’ performance. The final stage of incubation may have interfered with the embryo’s development, leading to this result. Avşar et al. [20] evaluated the effects of a higher eggshell temperature (38.6 °C) during the first 6 days of incubation and did not observe differences in the hatched chick weight in relation to the control group (37.8 °C). Leksrisompong et al. [60], evaluating higher egg temperatures (39.9 or 40.3 °C) on the 19th and 20th days of incubation, observed a reduction in the hatching weight and the relative weights of the organs of the hatching chicks. In the present study, high eggshell temperatures (42 °C, Table 3) on days 16, 17, and 18 of incubation for 24 h (T_24h_) did not influence the hatching weight or relative organ weight. It is necessary to better elucidate the relationship between this discrepancy in the literature and the embryonic period of TM.

## 5. Conclusions

The temperature during incubation can influence the performance and physiological characteristics related to thermotolerance in broiler chickens. On Days 16, 17, and 18 of incubation, raising the incubation temperature to 39 °C for 3 h may improve bird thermoregulation, but maintaining this temperature increase for 24 h impairs bird performance.

## Figures and Tables

**Table 1:** Environmental conditions (mean ± SD) during the rearing of broilers from eggs subjected to thermal manipulation during incubation.

Bird Age (Days)	Mean Temperature (°C)	Maximum Temperature (°C)	Minimum Temperature (°C)	Relative Humidity (°C)
1 to 7	28.8 ± 2.4	32.6 ± 1.2	23.5 ± 2.8	41.6 ± 7.4
7 to 14	25.5 ± 3.2	29.4 ± 2.1	22.2 ± 2.9	66.1 ± 20.3
14 to 21	24.5 ± 2.5	27.9 ± 1.7	21.2 ± 2.1	60.8 ± 17.8
21 to 28	23.9 ± 2.4	27.5 ± 2.1	20.0 ± 1.4	64.9 ± 12.7
28 to 35	25.5 ± 1.7	29.7 ± 1.6	20.5 ± 1.3	56.3 ± 8.7
35 to 42	23.9 ± 2.1	28.8 ± 1.8	20.4 ± 1.3	71.6 ± 10.2

**Table 2:** Proximate composition of diets for high-performance mixed broilers according to rearing phase.

Ingredient (%)	Bird Age (Days)			
	1 to 7	8 to 21	22 to 33	34 to 42
Corn	46.941	48.379	53.608	62.570
Soybean meal, 46% CP	45.754	43.489	37.988	30.334
Soybean oil	3.115	4.350	5.019	4.322
Dicalcium phosphate	1.997	1.724	1.417	1.075
Limestone	0.962	0.861	0.793	0.668
Salt (NaCl)	0.509	0.496	0.472	0.447
DL-Methionine, 99%	0.334	0.318	0.292	0.237
L-Lysine HCl, 78.8%	0.099	0.090	0.132	0.162
L-Threonine, 98%	0.034	0.033	0.039	0.026
Vitamin premix ^1^	0.100	0.100	0.080	0.060
Mineral premix ^2^	0.100	0.100	0.100	0.100
Coccidiostat ^3^	0.060	0.060	0.060	0.000
Total	100.00	100.00	100.00	100.00
Nutrient	Calculated composition			
Metabolizable energy (kcal/kg)	3000	3100	3200	3250
Calcium %	1.020	0.909	0.790	0.634
Available phosphorus %	0.486	0.434	0.369	0.296
Sodium %	0.221	0.215	0.206	0.196
Digestible protein %	22.560	21.700	19.730	17.105
Digestible methionine %	0.658	0.633	0.583	0.499
Digestible lysine %	1.355	1.293	1.192	1.033
Digestible threonine %	0.885	0.858	0.786	0.681
Digestible tryptophan %	0.296	0.283	0.253	0.212

^1^ Composition per kg of vitamin premix: folic acid (min.), 902.5 mg; pantothenic acid (min.), 12.0 g; biotin (min.), 77.0 mg; niacin (min.), 40.0 g; vitamin A (min.), 8,800,000.0 IU; vitamin B1 (min.), 2499.0 mg; vitamin B12 (min.), 16,200.0 mcg; vitamin B2 (min.), 5704.0 mg; vitamin B6 (min.), 3998.4 mg; vitamin D3 (min.), 3,000,000.0 IU; vitamin E (min.), 30,000.0 IU; vitamin K3 (min.), 2198.1 mg. ^2^ Composition per kg of mineral premix: copper (min.), 7000.0 mg; iron (min.), 50.0 g; iodine (min.), 1500.0 mg; manganese (min.), 67.5 g; selenium (min.), 349.6 mg; zinc (min.), 45.6 g. ^3^ Narasin.

**Table 3:** Hatchability, hatching weight, blood parameters, and organ weight of 1-day-old chicks from eggs subjected to thermal manipulation during incubation.

Variable	Treatment ^1^				SEM	*p*-Value
	Ctrl	T_3h_	T_12h_	T_24h_		
Incubator temperature (°C) ^2^	37.5 ± 0.1	39.0 ± 0.1	39.0 ± 0.2	39.0 ± 0.2	-	-
Shell temperature (°C) ^3^	40.2 ± 0.3	41.9 ± 0.2	42.1 ± 0.3	42.0 ± 0.1	-	-
Incubation time (h)	476.25	469.12	473.37	476.75	1.93	0.058
Hatching window (h)	43.50	50.75	46.37	43.00	4.75	0.652
Hatchability (%) ^4^	93.1 ± 1.7 ^a^	93.1 ± 1.7 ^a^	88.0 ± 2.2 ^b^	89.4 ± 2.1 ^b^	-	0.048
Hatching weight (g)	43.6	43.5	43.5	43.5	1.17	0.960
Glucose (mg/dL)	292.4	284.0	289.5	272.4	8.20	0.849
Hematocrit (%)	36.07	30.13	36.90	32.91	7.78	0.531
Corticosterone (ng/mL)	3.04	3.44	2.92	2.93	0.16	0.544
Triiodothyronine (ng/mL)	1.50	1.84	1.49	1.80	0.09	0.072
Thymus (%)	0.49	0.42	0.47	0.42	0.03	0.752
Bursa (%)	0.15	0.17	0.14	0.13	0.01	0.270
Spleen * (%)	0.05	0.03	0.04	0.04	0.00	0.737
Liver (%)	2.67	2.43	2.58	2.55	0.05	0.258
Heart * (%)	0.86	0.76	0.79	0.74	0.02	0.115
Small intestine (%)	4.83	3.90	4.64	4.57	0.19	0.408

^1^ Ctrl (control)—eggs incubated at 37.5 °C during the entire incubation period. T_3h_, T_12h_, and T_24h_—eggs incubated with an increase in incubation temperature to 39 °C on Days 16, 17, and 18 of incubation for 3 h/day, 12 h/day, and 24 h/day, respectively (*n* = 8). ^2^ Incubator temperature during thermal manipulation (mean ± SD). ^3^ Eggshell temperature during the half point of TM (mean ± SD). ^4^ Mean ± SD. * Log-transformed data. ^a,b^ Means followed by different letters in the row differ significantly by the Scott-Knott test (*p* < 0.05).

**Table 4:** Performance of broilers from eggs subjected to thermal manipulation during incubation.

Variable	Treatment ^1^				SEM	*p*-Value
	Ctrl	T_3h_	T_12h_	T_24h_		
Feed intake (g)						
1 to 21 days	1081 ^a^	1067 ^a^	1069 ^a^	1036 ^b^	10.7	0.048
1 to 33 days	2923 ^a^	2895 ^a^	2874 ^a^	2800 ^b^	27.2	0.029
1 to 42 days	4782 ^a^	4813 ^a^	4728 ^a^	4593 ^b^	53.6	0.049
Weight gain (g)						
1 to 21 days	876	875	878	835	14.5	0.142
1 to 33 days	2069	2065	2012	1979	33.3	0.211
1 to 42 days	2941 ^a^	2950 ^a^	2919 ^a^	2849 ^b^	26.8	0.047
Feed conversion						
1 to 21 days	1.24	1.22	1.22	1.25	0.05	0.458
1 to 33 days	1.42	1.41	1.43	1.42	0.03	0.844
1 to 42 days	1.63	1.63	1.62	1.62	0.02	0.889
Body weight (g)						
At 42 days	3030	3008	2973	2911	53.3	0.143

^1^ Ctrl (control)—eggs incubated at 37.5 °C during the entire incubation period. T_3h_, T_12h_, and T_24h_—eggs incubated with an increase in incubation temperature to 39 °C on Days 16, 17, and 18 of incubation for 3 h/day, 12 h/day, and 24 h/day, respectively (*n* = 8). ^a,b^ Means followed by different letters in the row differ significantly by the Scott-Knott test (*p* ≤ 0.05).

**Table 5:** Blood parameters, relative organ weight, intestinal morphometry, expression of heat shock protein 70 (HSP70) in the liver, carcass yield, and yield of cuts of broilers from eggs subjected to thermal manipulation during incubation.

Variable	Treatment ^1^				SEM	*p*-Value
	Ctrl	T_3h_	T_12h_	T_24h_		
At 21 days of age						
Glucose (mg/dL)	318.0	317.0	323.5	304.0	6.70	0.480
Hematocrit (%)	33.92	27.63	30.99	26.88	1.41	0.232
Total plasma protein (g/dL)	1.343	1.343	1.343	1.343	0.01	0.724
Corticosterone (ng/mL)	2.07	2.15	2.12	2.16	0.12	0.994
Triiodothyronine (ng/mL)	1.45	1.59	1.66	1.52	0.11	0.957
Thymus (%)	0.52	0.49	0.51	0.62	0.03	0.542
Bursa (%)	0.22	0.21	0.26	0.23	0.01	0.507
Spleen * (%)	0.08	0.09	0.09	0.09	0.01	0.636
Liver (%)	2.39	2.48	2.64	2.50	0.04	0.213
Heart * (%)	0.64	0.63	0.62	0.71	0.02	0.194
Intestine (%)	4.91	4.97	5.10	5.14	0.10	0.731
Villus height (µm)	1211	1120	1149	1203	48.9	0.805
Crypt depth µm)	215.85	216.93	207.37	196.7	6.69	0.487
Villus/crypt (µm/µm)	5.81	5.20	5.65	6.19	0.24	0.493
At 42 days of age						
Glucose * (mg/dL)	302.5	303.5	285.3	296.9	6.51	0.719
Hematocrit (%)	28.06 ^a^	24.27 ^b^	30.42 ^a^	25.91 ^b^	0.78	0.014
Total plasma protein (g/dL)	1.343	1.344	1.343	1.343	0.01	0.282
Corticosterone (ng/mL)	3.11	3.44	3.21	3.07	0.18	0.862
Triiodothyronine (ng/mL)	1.68	1.93	1.55	1.64	0.09	0.428
Thymus (%)	0.49	0.54	0.54	0.49	0.02	0.799
Bursa (%)	0.17	0.14	0.16	0.15	0.01	0.679
Spleen * (%)	0.11	0.10	0.10	0.10	0.01	0.610
Liver (%)	1.98	1.95	1.91	1.91	0.03	0.850
Heart * (%)	0.47	0.47	0.50	0.47	0.01	0.521
Intestine (%)	3.90	3.59	3.71	3.43	0.07	0.101
HSP70 in the liver	1.00 ^a^	1.64 ^b^	1.04 ^a^	0.92 ^a^	0.17	0.041
Heterophil (%)	30.5	38.87	32.25	30.2	7.83	0.988
Lymphocyte (%)	63.83	60.37	61.58	64.7	7.29	0.951
Heterophil/lymphocyte	0.51	0.54	0.52	0.50	0.19	0.998
Slaughter weight (g)	2.94	2.93	2.86	2.83	53.2	0.226
Carcass yield (%)	75.94	75.27	76.13	76.06	0.33	0.118
Breast yield (%)	36.93	39.40	38.36	38.41	0.41	0.231
Thigh + drumstick yield (%)	28.17	27.17	27.66	27.22	0.29	0.399

^1^ Ctrl (control)—eggs incubated at 37.5 °C during the entire incubation period. T_3h_, T_12h_, and T_24h_—eggs incubated with an increase in incubation temperature to 39 °C on Days 16, 17, and 18 of incubation for 3 h/day, 12 h/day, and 24 h/day, respectively (*n* = 8). * Log-transformed data. ^a,b^ Means followed by different letters in the row differ significantly by the Scott-Knott test (*p* < 0.05).

**Table 6:** Respiratory rate during the thermal challenge test of broilers obtained from eggs subjected to thermal manipulation during incubation.

Bird Age (Days)	Evaluation Time (ET)	Temperature (°C) ^1^	Relative Humidity (%) ^2^	Treatment (T) ^3^				SEM	*p*-Value		
				Ctrl	T_3h_	T_12h_	T_24h_		T	ET	T * ET
10	Before challenge	26.4 ± 0.3	59.6 ± 1.6	60.5	64.5	60.5	57.5	3.41	0.761	0.471	0.753
	Immediately after challenge	31.3 ± 0.4	57.4 ± 1.4	61.5	64.0	58.0	63.5				
	45 min after challenge	26.7 ± 0.3	59.9 ± 0.8	57.0	55.5	58.0	55.0				
17	Before challenge	25.2 ± 0.4	59.8 ± 0.9	63.0 ^a^	63.5 ^a^	63.5 ^a^	67.0 ^a^	3.22	0.635	0.002	0.849
	Immediately after challenge	29.9 ± 0.3	59.5 ± 0.8	67.5 ^a^	63.0 ^a^	61.0 ^a^	62.5 ^a^				
	45 min after challenge	25.2 ± 0.3	60.1 ± 0.8	56.5 ^b^	58.0 ^b^	53.5 ^b^	57.5 ^b^				
24	Before challenge	23.8 ± 0.3	60.0 ± 0.7	60.0	55.5	60.5	64.5	6.17	0.419	0.088	0.987
	Immediately after challenge	28.5 ± 0.3	59.6 ± 0.9	65.0	61.0	59.0	69.0				
	45 min after challenge	23.9 ± 0.4	59.9 ± 0.8	52.5	49.5	56.5	57.5				
31	Before challenge	24.8 ± 0.4	59.5 ± 1.6	55.0 ^Bb^	63.5 ^Ab^	58.5 ^Ab^	44.0 ^Bb^	8.14	0.013	0.001	0.938
	Immediately after challenge	29.5 ± 0.4	58.9 ± 1.3	79.5 ^Ba^	93.0 ^Aa^	94.0 ^Aa^	65.0 ^Ba^				
	45 min after challenge	24.8 ± 0.3	60.1 ± 0.7	50.5 ^Bb^	57.0 ^Ab^	59.5 ^Ab^	46.0 ^Bb^				

^1^ Chamber temperature during the thermal challenge tests (mean ± SD). ^2^ Relative humidity in the chambers during the thermal challenge tests (mean ± SD). ^3^ Ctrl (control)—eggs incubated at 37.5 °C during the entire incubation period. T_3h_, T_12h_, and T_24h_—eggs incubated with an increase in incubation temperature to 39 °C on Days 16, 17, and 18 of incubation for 3 h/day, 12 h/day, and 24 h/day, respectively (*n* = 8). ^a,b,A,B^ Means followed by different uppercase letters in the row and lowercase letters in the column within each age differ significantly according to the Scott-Knott test (*p* < 0.05).

**Table 7:** Cloacal temperature during the thermal challenge test of broilers obtained from eggs subjected to thermal manipulation during incubation.

Bird Age (Days)	Evaluation Time (ET)	Temperature (°C) ^1^	Relative Humidity (%) ^2^	Treatment (T) ^3^				SEM	*p*-Value		
				Ctrl	T_3h_	T_12h_	T_24h_		T	ET	T * ET
10	Before challenge	26.4 ± 0.3	59.6 ± 1.6	40.2	40.3	40.4	40.5	0.09	0.090	0.151	0.384
	Right after challenge	31.3 ± 0.4	57.4 ± 1.4	40.2	40.5	40.7	40.5				
	45 min after challenge	26.7 ± 0.3	59.9 ± 0.8	40.2	40.4	40.2	40.4				
17	Before challenge	25.2 ± 0.4	59.8 ± 0.9	40.8 ^a^	40.6 ^a^	40.5 ^a^	40.7 ^a^	0.08	0.791	0.001	0.952
	Right after challenge	29.9 ± 0.3	59.5 ± 0.8	40.9 ^a^	40.7 ^a^	40.7 ^a^	40.5 ^a^				
	45 min after challenge	25.2 ± 0.3	60.1 ± 0.8	40.4 ^b^	40.3 ^b^	40.3 ^b^	40.3 ^b^				
24	Before challenge	23.8 ± 0.3	60.0 ± 0.7	40.7 ^Aa^	40.6 ^Ba^	40.9 ^Aa^	40.8 ^A^	0.09	0.005	0.001	0.788
	Right after challenge	28.5 ± 0.3	59.6 ± 0.9	40.9 ^Aa^	40.8 ^Ba^	41.0 ^Aa^	40.9 ^A^				
	45 min after challenge	23.9 ± 0.4	59.9 ± 0.8	40.6 ^Ab^	40.4 ^Bb^	40.6 ^Ab^	40.8 ^A^				
31	Before challenge	24.8 ± 0.4	59.5 ± 1.6	41.0 ^a^	41.1 ^a^	41.2 ^a^	40.9 ^a^	0.07	0.088	0.001	0.967
	Right after challenge	29.5 ± 0.4	58.9 ± 1.3	41.1 ^a^	41.2 ^a^	41.4 ^a^	41.2 ^a^				
	45 min after challenge	24.8 ± 0.3	60.1 ± 0.7	40.8 ^b^	40.8 ^b^	40.9 ^b^	40.7 ^b^				

^1^ Chamber temperature during the thermal challenge tests (mean ± SD). ^2^ Relative humidity in the chambers during the thermal challenge tests (mean ± SD). ^3^ Ctrl (control)—eggs incubated at 37.5 °C during the entire incubation period. T_3h_, T_12h_, and T_24h_—eggs incubated with an increase in incubation temperature to 39 °C on Days 16, 17, and 18 of incubation for 3 h/day, 12 h/day, and 24 h/day, respectively (*n* = 8). ^a,b,A,B^ Means followed by different uppercase letters in the row and lowercase letters in the column within each age differ significantly according to the Scott-Knott test (*p* < 0.05).

**Table 8:** Metabolizability of dry matter, ash, lipids, and proteins by broilers obtained from eggs subjected to thermal manipulation during incubation.

Metabolizability Coefficient (%)	Treatment ^1^				SEM	*p*-Value
	Ctrl	T_3h_	T_12h_	T_24h_		
22–24 days of age *						
Dry matter	72.16	72.33	72.03	71.39	0.18	0.329
Ash	89.71	89.28	89.88	89.60	0.27	0.568
Lipids	90.79	90.40	90.68	90.57	0.19	0.830
Crude protein	65.15	66.19	65.03	66.07	0.46	0.724
35–37 days of age **						
Dry matter	74.04	74.86	74.22	74.20	0.52	0.898
Ash	88.95	88.90	89.12	89.03	0.21	0.970
Lipids	90.39	90.20	90.30	90.19	0.12	0.941
Protein	67.82	69.64	70.19	68.96	0.55	0.412

^1^ Ctrl (control)—eggs incubated at 37.5 °C during the entire incubation period. T_3h_, T_12h_, and T_24h_—eggs incubated with an increase in incubation temperature to 39 °C on Days 16, 17, and 18 of incubation for 3 h/day, 12 h/day, and 24 h/day, respectively (*n* = 8). * 22–24 days: maximum temperature, 28.3 °C; minimum temperature, 20.8 °C; and relative humidity, 62.25%. ** 35–37 days: maximum temperature, 29.7 °C; minimum temperature, 21.6 °C; and relative humidity, 64.94%.

## Data Availability

No new data were created or analyzed in this study. Data sharing is not applicable to this article.
